# Advances in the Experiments of Leaching in Cement-Based Materials and Dissolution in Rocks

**DOI:** 10.3390/ma16247697

**Published:** 2023-12-18

**Authors:** Lifan Zheng, Junjie Wang, Kefei Li, Mingyu Wang, Shimeng Li, Lin Yuan

**Affiliations:** Department of Civil Engineering, Tsinghua University, Beijing 100084, China; zlf21@mails.tsinghua.edu.cn (L.Z.); likefei@tsinghua.edu.cn (K.L.); wangmy21@mails.tsinghua.edu.cn (M.W.); sm-li21@mails.tsinghua.edu.cn (S.L.); yuan21@mails.tsinghua.edu.cn (L.Y.)

**Keywords:** leaching, rocks, cement-based materials, leaching mechanism, dissolution

## Abstract

Leaching in cement-based materials and dissolution in rocks are important problems in civil engineering. In the past century, concrete damage caused by leaching have occurred worldwide. And, rock dissolution is usually the main cause of karst rock erosions. This paper provides a review of the causes, influencing factors, and effects on engineering properties of dissolution of rocks and leaching of cement-based materials. The applied experimental methods for leaching and dissolution have been sorted out and discussed. In situ field experiments can be used to study dissolution under natural conditions, while the laboratory experiments can effectively shorten the experiment time length (by changing pH, temperature, pressure or other factors that affect the leaching or dissolution) to quickly investigate the mechanism of dissolution and leaching. Micro tests including XRD, SEM, EDS, and other testing methods can obtain the changes in material properties and microstructures under leaching and dissolution. In addition, with the advances in technologies and updated instruments, more and more new testing methods are being used. The factors affecting the leaching and dissolution include environmental factors, materials, and solvent parameters. The mechanisms and deterioration processes of leaching and dissolution varies according to the types of material and the compositions.

## 1. Introduction

Cement-based materials represented by concrete have a long history of thousands of years. For example, concrete was widely used in Roman structures founded from 300 B.C. to 476 A.D., which are still relatively intact today [1]. In 1824, Portland cement, mainly composed of silicate, was patented and made concrete an important material [1,2]. In civil engineering, leaching is often used to describe the corrosion of soluble components in concrete caused by water or other media [3]. People’s understanding of leaching has a long-time developing process. In the early 20th century, researchers recognized the risk of leaching and later Moskovin of the Soviet Union conducted a more systematic study [4]. Some scholars have pointed out that any concrete structure made of Portland cement must inevitably undergo the leaching of lime and lose all bonding properties within a certain period of time [5], and studies on leaching of concrete under different working conditions were conducted [6]. In the past 100 years, leaching of concrete has occurred frequently around the world, leading to an increasing emphasis on the leaching mechanism of concrete.

Dissolution in rocks refers to the process that soluble substances of rocks dissolve under the long-term action of solvents like rainwater, and the dissolved substances are lost, resulting in the formation of dissolution pores and cracks on the surface or inside of rocks, which commonly leads to karstification. Karstification is widely distributed worldwide, with a total karst area of 3.63 million kilometers in China. Among them, there are 2.03 million kilometers of exposed surface on carbonate rock, which is the largest contiguous exposed karst area in the world [7,8,9]. Karst and erosion not only brings many engineering problems [10] but also plays an important role in the actual projects and global carbon cycles [11,12,13]; in the area of oil and gas exploration in deep-buried layers, dissolution of rocks is also an urgent research issue [14,15,16]. During the 20th century, with the continuous increase in human engineering activities, karst disasters, as an engineering geological problem, were increasingly valued by the engineering community. In 1973, the International Association of Engineering Geosciences held an international conference in Hanover, Germany, titled “Karst Collapse and Settlement—Engineering Geological Issues Related to Soluble Rocks”. This was also the first international symposium on karst issues held by the engineering community [17]. In the 1990s, Chinese scholars compiled a map of soluble rock types in China and measured the distribution area of carbonate rocks in various provinces [18]. Afterwards, scholars from various countries around the world conducted extensive research on the causes, mechanisms, impacts, and establish mathematical models for dissolution, such as the process and mechanism of carbonate rock dissolution [19,20,21,22,23,24,25,26,27] and evaporites [28]. Some researchers also analyze and summarize karst phenomena under different types of materials or environmental conditions [29,30].

In this paper, we provide a review of experimental methods, mechanisms, and impact on engineering properties for dissolution in rocks and leaching in cement-based materials, and we analyze and compare the advantages of various methods in order to provide ideas and references for future research on experimental methods and conditions.

## 2. Experiment Methods for Leaching and Dissolution

### 2.1. Macroscopic Experiments

According to the external environment in which the experiment is conducted, macroscopic methods can be divided into two types: in situ field experiments and laboratory experiments.

#### 2.1.1. In Situ Field Experiments

**Figure 1:**
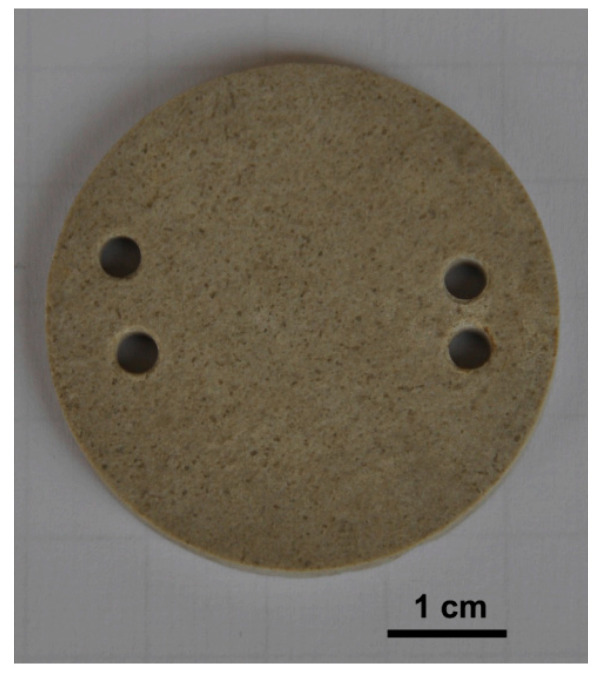
Limestone tablet [32].

In situ field experiments are often conducted using rock tablets. The sample is cut into standard-sized thin sheets, and is placed under different types of natural conditions, and researchers calculate the changes of various properties of the tablets for a period of time to characterize the dissolution [31,32]. The demonstration of rock tablets can be seen in Figure 1. Due to the difference in mineral composition between standard tablets and rocks excavated from local rock formations, rocks from the test site are usually used to produce tablets to obtain more accurate results [33]. But, the preparing conditions of the samples can also affect the results of the experiment [34]. Smooth tablets have lower dissolution rates than rough ones under the same conditions, and the lithology and altitude of the experiment area also have impacts. The totally weathered area of the tablets is larger than the surface area, as the dissolution occurs not only on the surface but also in the voids and cavities of the limestone tablet [35].

This method can be used in various karst experiments. For instance, Li. et al. cut the carbonate rocks in a certain area of Guangxi into thin sections and buried them in forest, shrubland, and farmland environments for a period of six months for natural dissolution, and they quantitatively studied the impact of drought of different durations on karst in three environments [36]. Yan Wei et al. buried the dolomite samples in the soil layers of dry land, paddy fields, and other areas for one year, obtained the dissolution rates of the samples under different land-use conditions, and analyzed the corresponding carbon sink intensity [37]. Wu Jianqiang et al. used the in situ field method to study the relationship between the dissolution rate of carbonate rocks and the content of calcium oxide in the southern Jiangsu region [38].

#### 2.1.2. Laboratory Simulation Experiment

**Figure 2:**
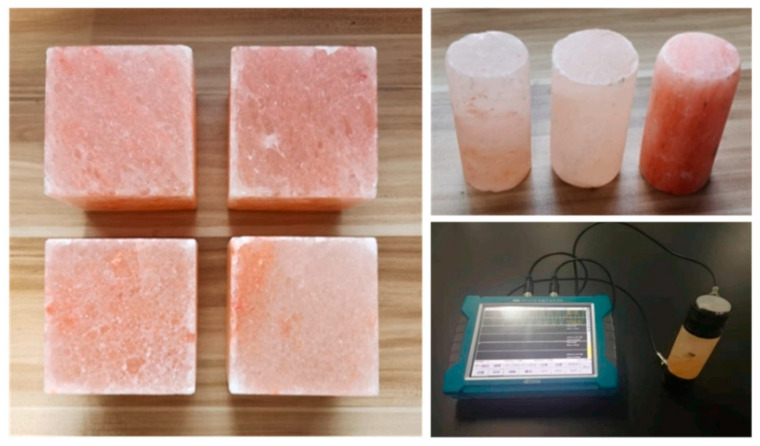
Rock salt specimens used in property and dissolution experiments [41].

Leaching and dissolution are relatively long processes, which usually take several years or decades under natural conditions to obtain obvious changes. Accelerated experiments are methods that accelerate leaching and dissolution by strengthening one or several influencing factors, which can obtain results in a short period of time to explore the mechanism. Experiments are usually conducted in groups, and some use equipment that can provide coupling experimental conditions [39,40]. A group of rock specimens used in a dissolution experiment is shown in Figure 2 as an example. Acidic reagents, deionized water, changing temperature, controlled solution flow rates, and other methods are commonly used, which can accelerate the process in a certain way.

The recent research conclusions and leaching or dissolution methods used in some literature can be seen in Table 1.

### 2.2. Microscopic Methods

It is hard to reveal the laws and mechanisms of leaching and dissolution only using macroscopic experimental methods; so, microscopic experimental methods are also needed to obtain information such as ion distribution, microscopic morphology, and identification of the reaction products. The main technical means of microscopic experiments include scanning electron microscopy (SEM), diffraction of X-rays (XRD), 3D laser scanning, energy dispersive spectroscopy (EDS), TG-DTA, ion concentration detection, etc.

**Figure 3:**
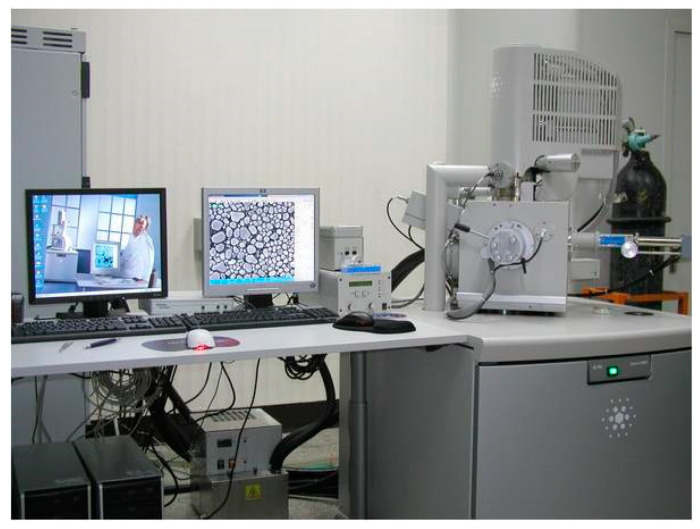
Scanning electron microscope.

**Figure 4:**
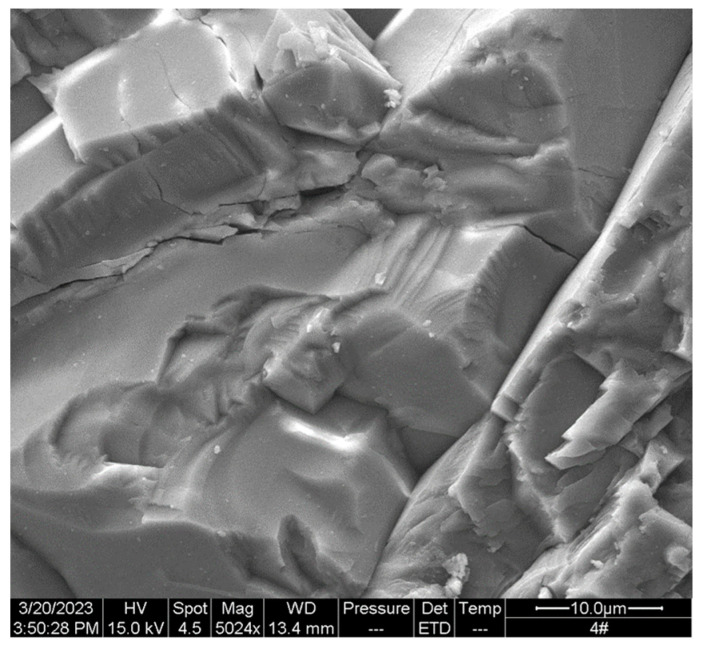
SEM image of white marble surface.

The SEM technology is to utilize a finely focused electron beam in a scanning electron microscope to bombard the specimen, getting various physical information through the interaction between electrons and the specimen. When applied to non-metallic materials, platinum or other conductive coatings are usually plated on the surface of the material. In leaching and dissolution research, SEM is often used to obtain the micro morphological characteristics of the surface of the test piece, such as the difference in the surface morphology of plastic cement-based materials before and after leaching [67], the micro morphology of C–S–H gel in the concrete test piece [53], and the development types and dissolution pore characteristics of rocks [68]. The SEM equipment is shown in Figure 3. Figure 4 shows the microscopic surface morphology of a white marble under SEM.

**Figure 5:**
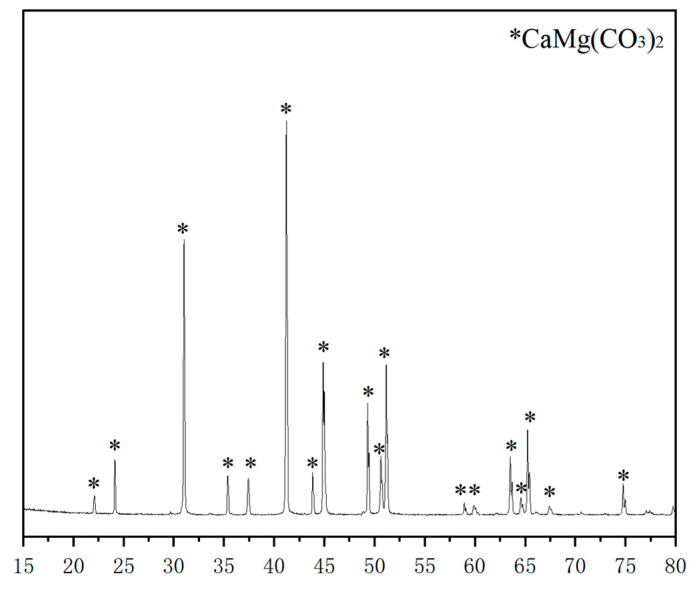
XRD diffractograms of a marble sample after dissolution.

The XRD technology can perform phase analysis of samples, and the changes in the relative content of a certain component before and after leaching can be analyzed through X-ray diffraction patterns [69]. For example, XRD can be used to compare the differences in mineral composition of sandstone cores before and after dissolution [70], study the order degree of rocks such as dolomite [71], and judge the differences in hydration products of cement [72]. Figure 5 shows the XRD diffractograms of a marble sample after dissolution; the chemical composition can be seen from the perspective and shape of the peaks.

Three-dimensional laser scanning uses high-speed laser and non-contact measurement to scan the target object in the area to obtain the 3D spatial point cloud dataset of the target object and convert it into a 3D spatial model in a unified coordinate state. Three-dimensional laser scanning has no contact with the specimen and is high-precision, which can accurately obtain accurate data on the surface corrosion morphology of the specimen. Three-dimensional laser scanning is widely used to provide the information about the surface morphology of exposed carbonate rocks [73,74,75,76], which can help to study the changes in porosity of rocks after dissolution [77] and can provide information about the dissolution rate and geometric evolution characteristics [78]. On this basis, 4D tomography has begun to be used to study the process of dissolution [79].

Due to the fact that various elements have their own X-ray characteristic wavelengths and the size of the characteristic wavelengths depends on the characteristic energy released during the energy level transition process, the EDS technology can utilize the different characteristic energies of X-ray photons from different elements for compositional analysis. It is often used in conjunction with other microscopic testing methods to determine the mineral composition of the sample. By combining EDS and SEM analysis methods, compared to traditional techniques such as XRD, testing objectives can be achieved at a lower cost and low mineral composition can be observed more easily [80].

The TG-DTA technology includes heating and records the quality changes of the sample during the testing process. The mass proportion of each component in the sample can be analyzed through the thermal analysis curve [81]. TG-DTA is used to record the relative content changes caused by the thermal decomposition of hydration products in concrete at a curing age of 28 days [82] and determine the hydration degree of recycled cement pastes [83].

The ion concentration detection can analyze the content of each ion in the leaching solution. For example, Mo Yunchuan [84] conducted ion composition testing on the solution leaked from various fractured limestone samples; Shutao Zhou used inductively coupled plasma mass spectrometry to determine the changes in elemental content of the solution during the leaching process [85].

The XCMT technology obtains digital images of material X-ray absorption from various angles and then obtains three-dimensional images via numerical reconstruction of a set of 2D images [86], which makes it an important technique to avoid damage to the microstructure of the sample.

**Figure 6:**
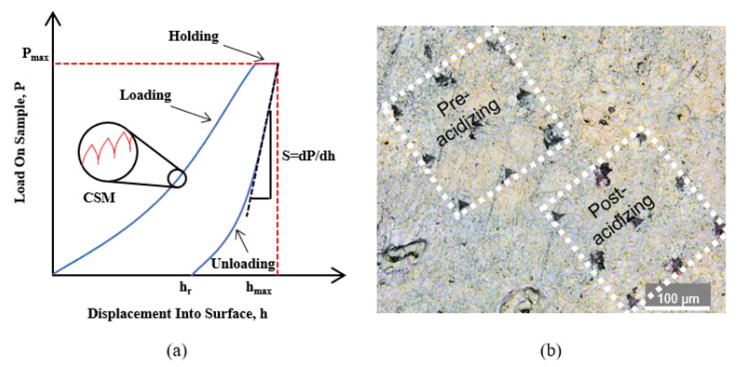
Nano indentation tests [91]. (**a**) Test result; (**b**) Test area

In addition, nuclear magnetic resonance (NMR) is also used to observe the pore size of porous rocks [87]; digital holographic microscopy is used in the nanoscale observations of the gypsum leaching in situ [88]; electron probe microanalysis (EPMA) is used to display significant differences between dissolution and non-dissolution areas to determine the boundaries [89]; a back-scattered electron (BSE) is used to measure in situ dissolution porosity of minerals [90]; and nano indentation tests are used to quantify the embedding strength of the fracture surface [91]. Figure 6 shows the test area and obtained test results; electron microprobe analysis is used to determine the exact composition of the samples [92]. CT Scanning is used to observe the spatial distribution of pores and fractures [93].

### 2.3. Simulation Experiment

With the improvement of numerical simulation software and computer performance, simulation experiments conducted using numerical simulation software such as COMSOL have also become more feasible and reliable. It is difficult to simulate the dissolution or leaching process using traditional in-situ or laboratory experiments due to with multiple factors or long-time spans, but simulation experiments can analyze the entire process. Under the reasonable selection of mechanism and setting of the influencing factors, such experiments can effectively simulate the development of leaching and dissolution in real situations. An independently developed evolutionary simulation software was used to conduct simulation experiments on the relationship between crack density, precipitation, and dissolution rate in carbonate rock mass [94]; the OpenSEES was applied to establish a finite element model of concrete column components considering leaching damage and obtain the laws of the changes in compressive and flexural bearing capacity of the components [95]; COMSOL was used to conduct numerical simulation tests on the leaching of cementitious materials in various parts of a certain reservoir dam body and compare them with actual monitoring results, analyzing the areas where the dam body is more severely affected [96]; PHEEQC was used to simulate the leaching of calcium carbonate under mixed conditions such as shallow groundwater, deep circulating hot water, and soil infiltration water [97] and can also be applied for modeling the geochemical reactions in porous media [98]; and three-dimensional numerical simulations were established to conducted multiple core displacement tests on carbonate rock cores [99].

## 3. The Mechanism of Leaching and Dissolution

### 3.1. The Dissolution of Rocks

The dissolution mechanism of rocks varies depending on the differences in their main components.

The dissolution of carbonate rocks is mainly the process of carbon dioxide dissolved in water and carbonates such as CaCO_3_ forming soluble substances such as calcium bicarbonates. Due to the fact that rocks are composed of particles of different sizes, dissolution is relatively strong in intergranular and intergranular pores, as well as in various types of fractures [100]. The dissolution of dolomite can be divided into four stages: the initial dissolution stage, secondary dissolution acceleration stage, stable dissolution rate stage, and dissolution attenuation stage; the dissolution rate of initial stage is much greater than the stable stage [101]. In addition, acidic solutions rich in hydrogen ions such as acid rain can also cause corrosion when in contact with carbonates [102,103]. The chemical reaction equation for acid-rain dissolution of carbonate rocks is as follows [104]. The meaning of the compounds can be found in Appendix A.

CaCO_3_ + H_2_SO_4_ → CO_2_ + Ca^2+^ + H_2_O + SO_4_^2−^ (1)

CaCO_3_ + 2HNO_3_ → CO_2_ + Ca^2+^ + H_2_O + 2NO_3_^−^ (2)

Among them, Yuan Daoxian proposed a karst dynamics model to visually represent the process [105], in which the environment was divided into three phases: gas phase, liquid phase, and solid phase. Carbon dioxide enters the liquid phase and reacts with water to produce hydrogen ions. Meanwhile, calcium carbonate dissolves into calcium ions and carbonate ions, and the latter reacts with the hydrogen ions to produce soluble bicarbonate ions.

Sulfate rocks, such as gypsum and anhydrite, have different dissolution mechanisms from carbonate rocks. Research has shown that even without the participation of carbon dioxide, water still has a certain solubility in sulfate rocks [106]. Besides chemical dissolution, physical effects also cannot be ignored [102]. At the same time, impurities such as calcite or dolomite in sulfate rocks such as gypsum also affect the equilibrium of dissolution, leading to changes in solubility [107]. 

Silicates are often difficult to dissolve in water due to their composition, and their dissolution can be used for carbonation and storage of carbon dioxide [108].

### 3.2. The Leaching of Cement-Based Material

**Figure 7:**
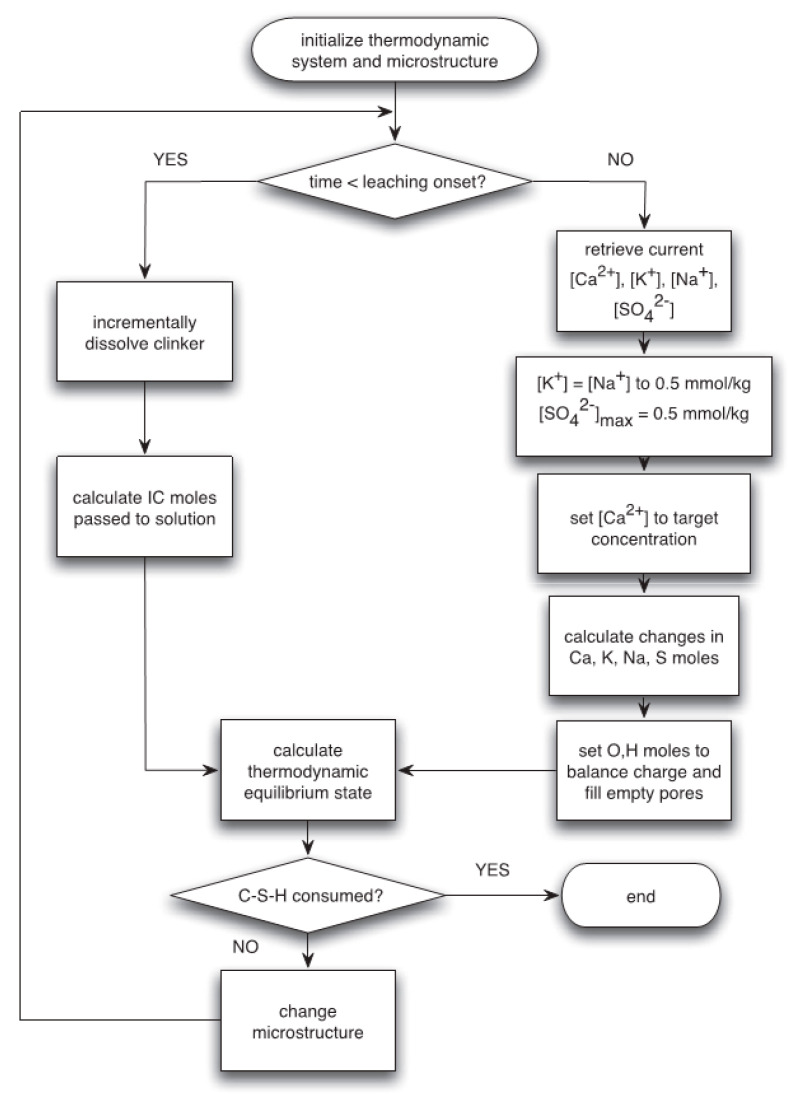
Flowchart of a leaching model for Portland cement binders [113].

The leaching of cement-based materials mainly comes from calcium dissolution. When there is a difference between the pore solution of cement-based materials and surrounding solvents such as deionized water, the concentration gradients break the initial solid–liquid equilibrium and induce the leaching of cement-based materials [109,110]. The solid phase calcium will dissolve into the pore solution to maintain the dissolution balance of calcium ions in the pore solution and keep pH stable. However, in soft water or acidic solution, the calcium hydroxide will continue to run off under the concentration gradient of the pore solution and the substances in the external environment, which will lead to the continuous decalcification and dissolution of C–S–H gel and ettringite, leading to the gradual decrease in calcium ion concentration in the pore solution, the constant decrease in pH value, and the gradual loss of gellability of the hydration products. Ultimately, it leads to material corrosion and damage. Among them, the calcium ion concentration in the pore solution of cement-based materials in soft water is higher than that in the environment, resulting in a difference in internal and external concentration, leading to continuous outward diffusion and leaching of calcium ions in the pores, which belongs to a physical process. In acidic solutions, the hydration products of cement react chemically with hydrogen ions, causing decalcification of cement-based materials, which belongs to a chemical process [53,111,112]. A flowchart of a leaching model for Portland cement binders is shown in Figure 7.

Ca(OH)_2_ → Ca^2+^ + 2OH^−^ (3)

nCaO·2SiO_2_·nH_2_O → nCa^2+^ + H_3_SiO4^−^ + [2n − 1]H_2_O (4)

or

Ca(OH)_2_ + 2H^+^ → Ca^2+^ + H_2_O (5)

C–S–H + 2H^+^ → Ca^2+^ + H_2_O + SiO_2_ (6)

C–A–H + 2H^+^ → Ca^2+^ + H_2_O + Al_2_O_3_ (7)

C_4_AF + 2H^+^ → Ca^2 +^ + H_2_O + Al_2_O_3_ + Fe_2_O_3_ (8)

## 4. The Impacts of Leaching and Dissolution on Material Properties

### 4.1. The Dissolution of Rocks

The impacts of dissolution on rocks are mainly reflected in the formation of the strength, dissolved pores, cracks, and surface roughness on the surface [103,114,115,116,117]. The components that can react with fluids will continuously decrease as the dissolution process progresses which leads to changes in rock mineral composition. And, the alteration in microstructure, pore characteristics, and other factors can also affect the physical and mechanical properties of rocks.

After dissolution, the porosity usually increases continuously, leading to the connection between pores in rocks [118,119], and the permeability of rocks may increase several times or even tens of times [116]. But, in some cases, the permeability of rocks may actually decrease. In a series of core flooding experiments, the calcite particles generated by dissolution blocked the pore channels in the rock core, resulting in a decrease in permeability [120]. In some cases, cracks appear in the rock. When reflected in the physical and mechanical properties of rocks, it is the increase in surface roughness and water absorption and decrease in strength [103,121]. The comparison on indicators of rocks before and after dissolution can be seen in Table 2.

### 4.2. The Leaching of Cement-Based Materials

C–S–H gel and Ca(OH)_2_ are important components to ensure the performance of concrete, which are closely related to the strength, density, and impermeability of cement-based material. When subjected to leaching, calcium precipitates and the microstructure changes, which leads to deterioration of the engineering properties [124,125]. The Comparison on indicators of cement-based materials before and after leaching can be seen in Table 3.

## 5. Discussion

The main experiment methods for leaching and dissolution are mainly divided into three types: laboratory experiments, in situ field experiments, and simulation experiments. Laboratory-accelerated experiments can significantly accelerate the process by strengthening one or several factors, thereby simulating the leaching or dissolution process and studying the reaction mechanisms. In situ field experiments were mainly used for long-term exposure tests. Simulation experiments like numerical modelling tests can be used when the related parameters were obtained. Leaching and dissolution are common forms of degradation in engineering, and there has been extensive research on the mechanism and influencing factors over the past hundred years. It can be seen that the mechanism of leaching and dissolution and the conditions for the formation have not been well understood and the related knowledge is constantly improved. The methods for conducting leaching and dissolution experiments are gradually becoming diversified and standardized. However, we should also note that, currently, most experiments are only conducted under one single experimental condition, and there are few experiments that study dissolution or leaching under multiple factors. In addition, there is a lack of research on the linking relationship between the experiment results conducted in the laboratory and the natural dissolution that occurs in the field.

The strengthening factors of laboratory-accelerated experiments mainly focus on several factors such as pH, temperature, and pressure, which are also the main factors affecting the dissolution rate and are relatively easy to change in an experiment. The factors affecting dissolution and leaching can be seen in Table 4 and Table 5. In addition, leaching and dissolution occurring in nature is often caused by the combined action of multiple factors. However, there are relatively few studies that involve the proportion of leaching or dissolution caused by one factor in the overall factors. If the factors selected in the experiment differ from the actual situation, it may lead to errors in the mechanism of leaching or dissolution research, which is also one of the problems that needs to be solved in the future. The influencing factors of rock dissolution and concrete leaching are slightly different, but there are many similarities.

**Figure 8:**
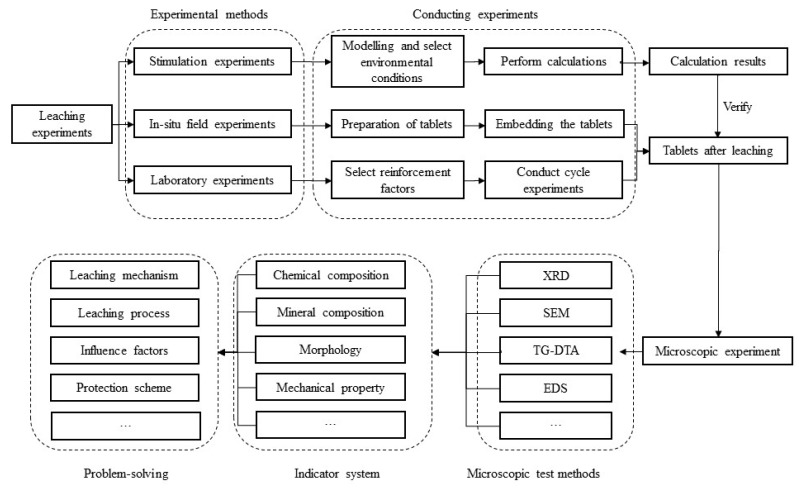
The flow chart of designing leaching and dissolution experiment.

The design of leaching and dissolution experiments can be carried out according to the following model shown in Figure 8. A leaching or dissolution experiment can be carried out using three types of methods, and the results coming from stimulation experiments can verify the results obtained from the other two kinds of experiments. Though microscopic experiment, the indicator needed by the problem can be obtained. 

There are many micro testing methods for leaching and dissolution, such as XRD, SEM, and EDS. Their purpose is focused on obtaining the chemical composition, mineral composition, surface morphology, porosity, and other purposes of the specimen. With the increasing demand for accuracy and functionality, more and more testing methods and instruments are being used in the research. At present, there are both common traditional testing methods such as XRD and SEM in research, as well as relatively new technologies put into use. In addition, the updates of portable testing instruments also provide conditions for on-site testing, which will greatly shorten testing time and facilitate the acquisition of experimental data from the test site. Overall, the new methods and traditional methods can be well combined to provide various information from multiple perspectives and with higher accuracy.

**Figure 9:**
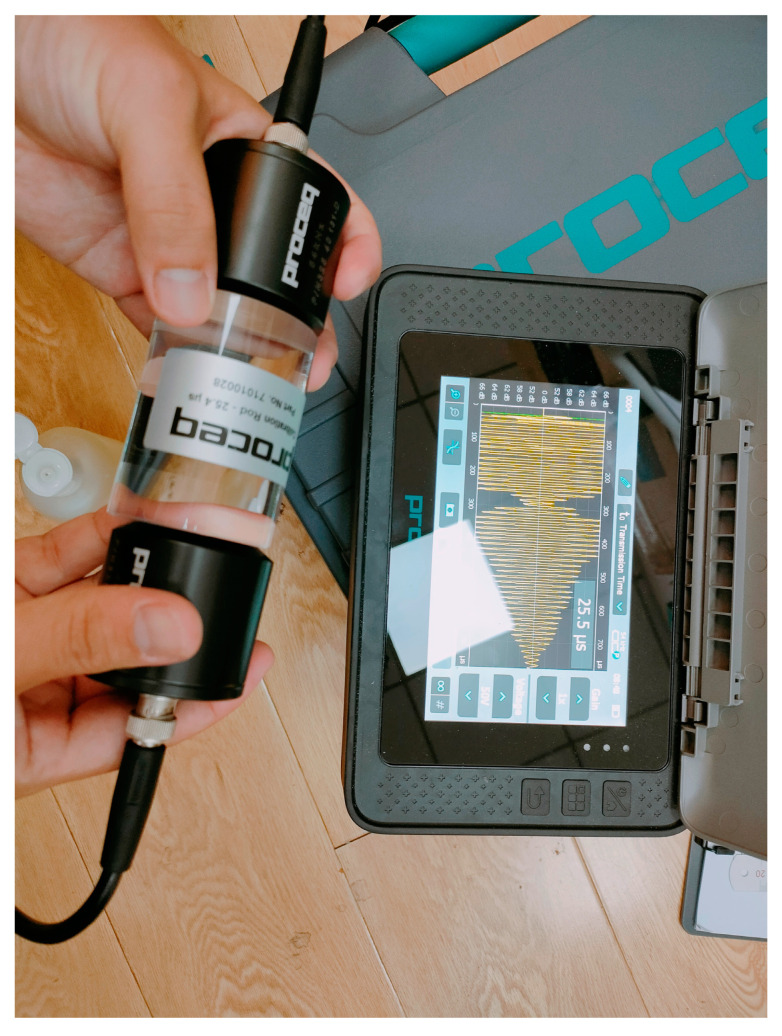
Ultrasonic non-destructive testing equipment.

At present, the widespread and complete application of concrete and rocks in the construction field is more inclined to indoor decoration. The research on the protection of existing stone buildings is also receiving increasing attention. Therefore, the testing methods for rocks should be combined with quasi non-destructive testing or non-destructive testing, such as ultrasonic testing, 3D laser scanning, etc. For example, ultrasonic non-destructive testing is a convenient, fast, and inexpensive testing method like in Figure 9. Through two ultrasonic transmitting and receiving devices, researchers can quickly obtain information such as wave velocity and waveform to determine changes in material properties. This is a testing method worth promoting, but currently there is still room for improvement in ultrasonic testing instruments for non-metals.

## 6. Conclusions

This study investigated the factors influencing the leaching of cement-based materials and leaching of rocks, the related experiment methods were discussed and the effect of leaching and dissolution on the engineering properties were reported. The following conclusions can be drawn:

(1) There are three main types of leaching and dissolution experiments: laboratory experiments, field in situ experiments, and simulation experiments. Field in situ experiments can obtain more accurate results under real conditions but are relatively difficult to conduct; laboratory experiments are usually accelerated experiments that can fasten the entire process in a short period of time; simulation experiments can simulate long-term and dynamic processes but require careful selection of simulation environments and conditions, which are usually used as auxiliary means for other experiments.
(2) XRD, SEM, and other testing methods are widely used in the study of dissolution and leaching. With the advancement in technology, more high-precision and high-resolution technologies have been developed, making the indicators of dissolution more adequate and accurate.
(3) There are many factors that affect leaching of cement-based materials and rock dissolution, including environmental factors, material properties, and solvent properties. These influencing factors often serve as the key for designing an accelerated experiment.
(4) The mechanism of rock dissolution varies depending on its mineral composition. Typically, rocks containing more carbonate rock components are more prone to dissolution.
(5) The leaching of cement-based materials is mainly due to the dissolution of calcium. During the process, the hydration products gradually lose their intact structure, leading to corrosion and destruction.
(6) Leaching can cause deterioration of engineering structural properties. Changing the compositions of the material can alter the process of deterioration, which is also an important direction for optimizing cement-based materials.

## Figures and Tables

**Table 1:** Some leaching and dissolution methods and research conclusions.

Reinforcing Factor	Research Conclusion
High temperature and high pressure	Simulated the leaching effect of dolomite reservoirs under burial conditions and explored the dissolution laws of different types of dolomites [42].
pH and high temperature	pH affects the selective dissolution of feldspar and calcite, and the existing species of silicon and calcium in solution varies depending on the pH value [43].
CO_2_ concentration	The average total dissolution rate of rock samples in an open environment is 1.43 times that of a semi-open one and 2.70 times that of a closed area. The CO_2_ exchange reaction is the main influencing factor [44].
High temperature, high pressure, and pH	Under mixed deposition conditions, the main dissolved minerals are different at different temperature ranges [45].
Temperature and pressure	The development of buried dissolution pores are controlled by rock physical properties, acidic fluid concentration, and temperature [46].
High temperature and high pressure	The injection of acidic fluids into deep open systems caused by fracture opening or activation can lead to partial dissolution of dolomites, and the scale is related to fluid flux [47].
Deionized water	Simulations were conducted to investigate the contact leaching characteristics of three types of materials, namely cement slurry, cement fly ash, and cement slag, under the action of deionized water using relatively small water-to-solid ratios and different surface areas [48].
pH	Through uniaxial compression tests of the limestone at different angles and directions, the characteristics of limestone under dissolution were studied [49].
pH	Studied the time-varying laws of three evaluation indicators, including tensile strength of layer splitting, dissolution depth, and cumulative relative dissolution of calcium ions [50].
Deionized water	Studied the influence of mineral admixtures such as fly ash and slag on the leaching performance of cement slurry in deionized water and their dosage [51].
pH	The effect of sulfuric acid action on the leaching rate of cement mortar was studied, and the curve of the change in acid consumption of the specimen with immersion time was obtained [52].
pH	The mechanism and microscopic morphology of calcium leaching in cement-based materials [53].
Temperature, pressure, and pH	Deriving the dissolution rate of sandstone at different scales using vertical scanning interferometry [54].
pH and deionized water	Ammonium nitrate solution can effectively simulate and accelerate the leaching process of dam concrete, and the driving force for accelerating leaching is the acid-base neutralization reaction between ammonium nitrate and cement hydration products to produce highly soluble calcium nitrate [55].
pH	Under stress, the solid reaction activation energy of the solid–liquid contact surface in the reaction system increases. Stress causes deformation of the rock mass, and cracks on the surface of the rock mass continue to expand and extend under stress, increasing the solid–liquid contact area and promoting the occurrence of dissolution [56].
High temperature, high pressure, and pH	Under open system high-temperature and high-pressure conditions, organic acid dissolution of feldspar components in sandy conglomerate can effectively improve reservoir porosity and permeability [57].
Water pressure and temperature	The dissolution of carbonate rocks includes chemical and physical dissolution. As the hydrodynamic pressure increases, the ratio of simultaneous increase in chemical and physical dissolution damage tends to be the same. There is a coupling relationship between chemical dissolution and physical damage [58].
pH (controlled by the dissolution of CO_2_)	Changes in porosity and permeability of carbonate rocks immersed in water containing carbon dioxide were studied [59].
Temperature	The direct relationship between the dissolution rate of limestone and temperature was studied [60].
Temperature, high pressure, PH	Saltwater flow and carbon dioxide in carbonate rock cores promote dissolution, leading to an increase in porosity [61].
High temperature, high pressure, and pH	Lithology, fluid type, and temperature and pressure conditions can all affect dissolution. Gypsum is more soluble than limestone and dolomite. The type of fluid has almost no effect on the dissolution of gypsum rock [62].
pH	Leaching experiments and uniaxial compressive strength tests were performed to analyze the karst development characteristics and mechanism of concrete leaching [63].
Temperature and pressure	Simulate the dissolution process of carbonate rocks [64].
pH	Study the influence of chemical and mineralogical composition on dissolution of phosphate rock samples [65].
Temperature	The diffusion coefficient of concrete is positively correlated with temperature and negatively correlated with the degree of hydration. The type of cement has an impact on the diffusion coefficient, while the water–cement ratio has no significant relationship with the diffusion coefficient [66].

**Table 2:** Comparison on indicators of rocks before and after dissolution.

The Type of Rocks	Engineering Property Indicators	Before Dissolution	After Dissolution	Conclusions
Dolomite Marble	Porosity	1.1%	4.7%; 7.2%; 8.2%	After dissolution, the porosity and pore size of the rock significantly increased. The development of dissolution pores and gaps occurred on the surface of the rock [122].
Dolomite Marble	Pore radius	0.05~0.32 μm	3~5 μm	
Limestone	Compressive strength	115.2 MPa	129.6 Mpa	After dynamic dissolution experiments on limestone, the compressive strength of the rock decreased by 18% and the Young’s modulus reduced by 26% [123].
Limestone	Elastic modulus	26,585.5 Mpa	19,754.3 Mpa	
Mixture of dolomite, calcite, and quartz	Poisson’s ratio	0.176	0.116	The rock became more easily broken under the stress after dissolution. Additionally, the Poisson’s ratio also decreased by 34.09% due to salt dissolution. Therefore, salt dissolution overall reduced the rock strength of saline-lacustrine carbonate rock to some extent [116].
Mixture of dolomite, calcite, and quartz	Mineral composition	88.99% calcite, 3.17% dolomite, and 7.58% quartz	79.61% calcite, 9.68% dolomite, and 9.27% quartz	This variation in the mineral composition suggested that calcite in the carbonate samples reacted during the dissolution experiment, while the other two components had almost no reaction [117].

**Table 3:** Comparison on indicators of cement-based materials before and after leaching.

The Type of Cement-Based Materials	Engineering Property Indicators	Before Leaching	After Leaching	Conclusions
Calcium silicate hydrates	The Ca/Si atomic ratio	0.91	0.02	The leaching of cement-based materials mainly resulted in the loss of calcium, and the increase in the specific surface area of C–S–H(I) samples after leaching was mainly attributed to the modification of pore structure, which was caused by the transformation of two-dimensional structure of C–S–H(I) into C–S–H with cross-linking structure and silica gel with three-dimensional structure [53].
Calcium silicate hydrates	Specific area	<200	>275	
Fly ash concrete	Compressive strength	15 Mpa	7.5 Mpa	The compressive strength and elastic modulus of concrete with different fly ash dosages had a similar trend with leaching time, and the compressive strength continuously decreased, with the most obvious decrease in the strength of concrete without fly ash added [126].
Fly ash concrete	Elastic modulus	4500	About 3000	
Silicate concrete	Pore volume	27~30%	35~37%	There was a correlation between the dissolution of portlandite and the increase in the volume of pores larger than 200 nm [127].
Silicate concrete	Tensile strength	3.0~3.25	2.5~2.75	After leaching, all fracture property parameters rapidly decreased, including the crack initiation load,maximum load, fracture toughness, elastic modulus, tensile strength, and fracture energy [128].

**Table 4:** Factors affecting dissolution of rocks.

Type	Influence Factor
Environmental factor	Temperature
	Pressure
	Gas phase components
	Illumination
Solvent	Solvent components
	pH
Material properties	Composition
	Mineral composition
	Pore characteristics
	Surface roughness

**Table 5:** Factors affecting leaching of cement-based materials.

Type	Influence Factor
Environmental factor	Temperature
	Pressure
	Gas-phase components
	Illumination
Solvent	Solvent components
	pH
Material properties	Type of cement
	Aggregate
	Water–cement ratio
	Pore characteristics
	Stress state

## Data Availability

Data are contained within the article.

## Appendix A

Compound	Identification
CaCO_3_	Calcium carbonate
H_2_SO_4_	Sulfuric acid
HNO_3_	Nitric acid
Ca^2+^	Calcium ion
SO_4_^2-^	Sulfate ion
NO_3_^-^	Nitrate ion
Ca(OH)_2_	Calcium hydroxide
OH^-^	Hydroxide ion
nCaO·2SiO_2_·nH_2_O	Calcium silicate
C-S-H	Hydrate calcium silicate
C-A-H	Calcium aluminium hydrate
C_4_AF	Tetra calcium aluminoferrite
